# Localization of Retinal Ca^2+^/Calmodulin-Dependent Kinase II-β (CaMKII-β) at Bipolar Cell Gap Junctions and Cross-Reactivity of a Monoclonal Anti-CaMKII-β Antibody With Connexin36

**DOI:** 10.3389/fnmol.2019.00206

**Published:** 2019-08-28

**Authors:** Stephan Tetenborg, Shubhash Chandra Yadav, Bianca Brüggen, Georg R. Zoidl, Sheriar G. Hormuzdi, Hannah Monyer, Geeske M. van Woerden, Ulrike Janssen-Bienhold, Karin Dedek

**Affiliations:** ^1^Animal Navigation/Neurosensorics, Institute for Biology and Environmental Sciences, University of Oldenburg, Oldenburg, Germany; ^2^Department of Biology & Center for Vision Research, York University, Toronto, ON, Canada; ^3^Division of Neuroscience, Ninewells Hospital and Medical School, University of Dundee, Dundee, United Kingdom; ^4^Cancer Research Center (DKFZ), Heidelberg, Germany; ^5^Department of Neuroscience, Erasmus MC University Medical Center, Rotterdam, Netherlands; ^6^Department of Neuroscience, Visual Neuroscience, University of Oldenburg, Oldenburg, Germany; ^7^Research Center Neurosensory Science, University of Oldenburg, Oldenburg, Germany

**Keywords:** CaMKII, gap junction, electrical synapse, connexin36, bipolar cell, retina, cross-reactivity, antibody

## Abstract

Neuronal gap junctions formed by connexin36 (Cx36) and chemical synapses share striking similarities in terms of plasticity. Ca^2+^/calmodulin-dependent protein kinase II (CaMKII), an enzyme known to induce memory formation at chemical synapses, has recently been described to potentiate electrical coupling in the retina and several other brain areas *via* phosphorylation of Cx36. The contribution of individual CaMKII isoforms to this process, however, remains unknown. We recently identified CaMKII-β at electrical synapses in the mouse retina. Now, we set out to identify cell types containing Cx36 gap junctions that also express CaMKII-β. To ensure precise description, we first tested the specificity of two commercially available antibodies on CaMKII-β-deficient retinas. We found that a polyclonal antibody was highly specific for CaMKII-β. However, a monoclonal antibody (CB-β-1) recognized CaMKII-β but also cross-reacted with the C-terminal tail of Cx36, making localization analyses with this antibody inaccurate. Using the polyclonal antibody, we identified strong CaMKII-β expression in bipolar cell terminals that were secretagogin- and HCN1-positive and thus represent terminals of type 5 bipolar cells. In these terminals, a small fraction of CaMKII-β also colocalized with Cx36. A similar pattern was observed in putative type 6 bipolar cells although there, CaMKII expression seemed less pronounced. Next, we tested whether CaMKII-β influenced the Cx36 expression in bipolar cell terminals by quantifying the number and size of Cx36-immunoreactive puncta in CaMKII-β-deficient retinas. However, we found no significant differences between the genotypes, indicating that CaMKII-β is not necessary for the formation and maintenance of Cx36-containing gap junctions in the retina. In addition, in wild-type retinas, we observed frequent association of Cx36 and CaMKII-β with synaptic ribbons, i.e., chemical synapses, in bipolar cell terminals. This arrangement resembled the composition of mixed synapses found for example in Mauthner cells, in which electrical coupling is regulated by glutamatergic activity. Taken together, our data imply that CaMKII-β may fulfill several functions in bipolar cell terminals, regulating both Cx36-containing gap junctions and ribbon synapses and potentially also mediating cross-talk between these two types of bipolar cell outputs.

## Introduction

Electrical synapses in the nervous system provide a fast route for intercellular signal transmission and fulfill several unique functions such as cell synchronization and network oscillations (Hormuzdi et al., 2001; Christie et al., 2005). Structurally, electrical synapses are gap junctions made of connexin proteins which belong to a gene family that comprises 20 different isoforms in the mouse. Amongst these isoforms, connexin36 (Cx36) is considered to be the main neuronal connexin due to its high abundance in the central nervous system (CNS), especially in the cerebellum, the olfactory bulb and the retina, where it couples a variety of different cell types (Bloomfield and Völgyi, 2009). The vast majority of retinal Cx36 is expressed in AII amacrine cells (Feigenspan et al., 2001; Meyer et al., 2014), which integrate rod-generated signals and transmit them *via* gap junctions into the cone pathway to enable scotopic vision (Güldenagel et al., 2001; Deans et al., 2002). Apart from the AII amacrine cell, Cx36 was also identified in photoreceptors (Feigenspan et al., 2004; Bolte et al., 2016), bipolar cells (Feigenspan et al., 2004; Han and Massey, 2005), ganglion cells (Schubert et al., 2005; Pan et al., 2010), and other amacrine cells (Brüggen et al., 2015; Yadav et al., 2019).

Accumulating evidence suggests that electrical and chemical synapses share striking similarities in terms of plasticity and may be regulated by the same key molecules (Pereda, 2014; Miller and Pereda, 2017; Alcamí and Pereda, 2019). Ca^2+^/calmodulin-dependent protein kinase II (CaMKII), an enzyme known to induce memory formation, is capable of potentiating electrical coupling in an activity-dependent manner (Alev et al., 2008; del Corsso et al., 2012). This mechanism is quite conserved among species (e.g., rabbit: Kothmann et al., 2012; goldfish: Pereda et al., 1998; mouse: Turecek et al., 2014) and relies on activation of glutamatergic synapses that are situated in close proximity to neuronal gap junctions. Excitation of glutamate receptors in these synapses produces a Ca^2+^ influx that drives CaMKII activation and subsequent phosphorylation of Cx36, thereby enhancing electrical coupling (Alev et al., 2008; Flores et al., 2010; Kothmann et al., 2012). Recent reports indicate that this pathway operates in Mauthner cells in teleosts (Yang et al., 1990; Flores et al., 2010), neurons of the mammalian inferior olive (Turecek et al., 2014), and AII amacrine cells of the mammalian retina (Kothmann et al., 2012), suggesting that CaMKII is a well conserved and essential regulator of neuronal gap junctions in different tissues and vertebrate classes.

Although CaMKII is considered a key molecule of synaptic plasticity, the role of its different isoforms in modulating electrical synapses remains unknown. Here, we studied the cell types that contained Cx36 gap junctions and also expressed CaMKII-β in the inner plexiform layer (IPL) of the mouse retina. Using a polyclonal antibody, we identified CaMKII-β expression in a subset of bipolar cell terminals and revealed that CaMKII-β was mainly confined to gap junctions of HCN1-positive type 5 bipolar cells but was also present in putative type 6 bipolar cells. Likely, the expression of Cx36 at these synapses does not depend on the β-subunit as CaMKII-β deficiency did not alter the size and number of Cx36 puncta. Also, CaMKII-β localization was not restricted to gap junction plaques but filled large parts of the bipolar cell terminal. Taken together, our data suggest that CaMKII-β may be involved in plastic changes at gap junctions in the terminals of type 5 bipolar cells and may also strongly affect glutamate release at these terminals.

## Materials and Methods

### Animals and Tissue Preparation

All procedures were approved by the local animal care committee (*Niedersaechsisches Landesamt fuer Verbraucherschutz und Lebensmittelsicherheit*) and were in compliance with the guidelines for the welfare of experimental animals issued by the European Communities Council Directive of 24 November 1986 (86/609/EEC) and the laws of the Federal Government of Germany (*Tierschutzgesetz*; BGBl. I S. 1206, 1313 and BGBl. I S. 1934). The experiments in this study were conducted with C57BL/6J, Cx36+/− Cx36-EGFP mice (comparable to Cx36+/+ Cx36-EGFP mice, see Meyer et al., 2014), CaMKII-β^+/+^ and CaMKII-β^−/−^ mice (Kool et al., 2016), and Ier5-GFP mice (Siegert et al., 2009; Yadav et al., 2019). Mice were deeply anesthetized with CO_2_ and killed by cervical dislocation. Eyes were enucleated and dissected in phosphate buffered saline (PBS). The cornea was removed by cutting around the ora serrata. Afterward, the lens and vitreous were removed and the eyecups were fixed in 2%–4% paraformaldehyde in 0.1 M phosphate buffer (PB) for 20 min.

### Constructs and N2A Cell Transfections

Transfection of N2A cells with Cx36 was carried out as previously described in Meyer et al. (2016). Twenty-four hours prior to transfection 1 × 10^5^ N2A cells were plated on 6 cm dishes in 5 ml Dulbecco’s Modified Eagle Medium (Biochrom GmbH, Berlin, Germany), supplemented with 10% fetal bovine serum (Biochrom). For transfection, 5 μg of DNA was mixed with the precipitation solution and applied to N2A cells. Cells were lysed or fixed 48 h after transfection.

### Immunohistochemistry and Immunocytochemistry

Eyecups were cryoprotected in 30% sucrose in 0.1 M PB and embedded in TissueTek. Afterward, the retina was cut into 20 μm sections which were blocked with 10% normal goat (NGS) or normal donkey serum (NDS) in Tris-buffered saline (50 mM Tris/HCl, 1.5% NaCl, 0.3% Triton X-100, pH 7.4, TBS-Tx). Primary antibodies were diluted in blocking solution and incubated over-night at 4°C. The following day, slices were washed in TBST-Tx and incubated with the secondary antibodies (conjugated to either Alexa488, Alexa568, or Alexa647, Thermo Fisher Scientific, Waltham, MA, USA) for 2 h at room temperature. Afterward, sections were washed with TBS-Tx and mounted in Aqua Polymount.

Immunostainings on retinal whole-mounts were conducted similarly, but primary and secondary antibodies were applied for 2–3 days and 1 day, respectively, at room temperature. After thorough washing, retina pieces were mounted in Vectashield.

For immunocytochemistry, a slightly different protocol was used. Cells were fixed in 2% paraformaldehyde in 0.1 M PB for 15 min at room temperature. After fixation and extensive washing with 0.1 M PB, cells were incubated in blocking solution containing 10% NGS in 0.1 M PB + 0.5% Tx100. Primary antibodies were diluted in blocking solution and incubated overnight. Secondary antibodies were incubated for 2 h at room temperature. Coverslips were mounted in Vectashield.

### Intracellular Dye Injections

Alexa Fluor 568 potassium hydrazide was injected into AII and A8 amacrine cells in C57BL/6J and Ier5-GFP mouse retina, respectively, as described earlier (Meyer et al., 2018; Yadav et al., 2019). Retinal pieces from C57BL/6J mice were incubated in 0.0005% DAPI solution for 45–60 min ahead of dye injections in order to visualize AII cell somata. In brief, sharp microelectrodes were pulled with a Sutter P-97 puller (Sutter, Novato, CA, USA) and typically had a resistance of 100–200 MΩ. The microelectrode tips were first filled with 2 μl of 10 mM Alexa Fluor 568 potassium hydrazide and afterward backfilled with 8 μl of 0.2 M KCl. To label AII/A8 cells, the dye was iontophoresed with a small negative current (-1 nA, square pulses of 500 ms at 1 Hz) for 5 min. Thereafter, the dye was allowed to diffuse for 15 min ahead of the fixation.

### Image Acquisition and Analysis

Images were acquired with a confocal laser scanning microscope (Leica TCS SP8). Retinal sections and whole-mounts were scanned with an HC PL APO CS2 63×/1.4 and 40×/1.3 objective, respectively. The pixel size was adjusted for each experiment and kept constant between different conditions. Step size in Z direction was 0.16–0.5 μm. Image stacks were deconvolved with Huygens Essential deconvolution software. Unless stated otherwise, single confocal scans are shown.

Stacks were adjusted for contrast and brightness in Fiji^1^ (Schindelin et al., 2012). For presentation purposes, some images were saturated in brightness to allow for better visibility of the small immunoreactive structures. For colocalization analyses saturation was avoided.

Quantification of Cx36-immunoreactive puncta expressed on bipolar cell terminals was performed with the *Colocalization Highlighter* plugin in Fiji. Both image stacks (six confocal scans, 0.2 μm each) were thresholded using the* Auto Threshold* function. Colocalized puncta were maximum projected. A region of interest (ROI, 23 × 5.4 μm^2^) was placed at the border between OFF and ON layers and puncta were measured in terms of frequency and size. Puncta smaller than 8 pixels^2^ were excluded from quantification.

Quantification of immunostainings in retinal whole-mounts was performed as described recently (Yadav et al., 2019), with slight modifications: in a ROI (48 × 48 × 5 μm^3^), we first estimated colocalization for CtBP2 and Cx36, using the *Colocalization Highlighter* plugin in Fiji. The resulting colocalized puncta were checked for additional colocalization with CaMKII-β. Colocalization was then checked by sequential 3D-rotation of the stack as described in Yadav et al. (2019). Please note that the colocalization obtained by using the *Colocalization Highlighter* was only slightly overestimated when compared to the 3D-rotation analysis. As it was not feasible to apply the 3D-rotation analysis to the hundreds of CtBP2 and Cx36 puncta present in the ROI, we carried out this analysis only for the triple colocalization as the numbers were expected to be much smaller.

### Protein Purification and Immunoprecipitation and Western Blot

Plasmids encoding the intracellular domains of Cx36 were cloned into pGEX6P2 vector, allowing for N-terminal tagging with GST. Proteins were expressed in BL21 cells. Expression was induced with 1 mM isopropyl-β-D-thiogalactopyranoside. After expression, cells were lysed with lysozyme and insoluble material was removed by centrifugation at 17,000 rpm for 40 min at 4°C. The supernatant was applied to a glutathione sepharose column and incubated overnight at 4°C. After extensive washing with binding buffer [containing 150 mM NaCl, 50 mM Tris (pH 7.4), 10 mM dithiothreitol], GST-fusion proteins were eluted from the column with elution buffer [containing 150 mM NaCl, 50 mM Tris (pH 7.4), 10 mM dithiothreitol and 10 mM reduced glutathione]. Immunoprecipitation (IP) was carried out as previously described in Meyer et al. (2016). For crosslinking of CB-β-1, 1–2 μg of antibody were applied to 50 μl magnetic protein G beads (Miltenyi Biotec GmbH, Bergisch Gladbach, Germany) and incubated for 15 min in 20 mM dimethyl pimelimidate (diluted in tri-ethonalamine, pH 8). Retinas were dissected as described above and homogenized in IP buffer, containing 0.5% NP-40, 50 mM Tris (pH 7.4), 150 mM NaCl and phosphatase and protease inhibitors (Roche Diagnostics, Mannheim, Germany). The lysate was incubated for 1 h on ice and centrifuged for 10 min at 10,000 *g* at 4°C. The supernatant was removed and precleared with protein G beads. Afterward, the lysate was incubated with previously prepared CB-β-1 beads for 2 h. After several washes, proteins were eluted with elution buffer, containing 50 mM Tris HCl (pH 6.8), 50 mM dithiothreitol, 1% SDS, 1 mM EDTA, 0.005% bromophenol blue, and 10% glycerol. SDS-PAGE (10% gels) and western blot were performed with homogenates and IP samples. Enhanced chemiluminescence was carried out as previously described in Meyer et al. (2016). Nitrocellulose membranes were blocked in 5% milk powder in TBS-Tween (20 mM Tris/HCl, 150 mM NaCl, 0.2% Tween, pH 7.4) and incubated with the respective antibody overnight at 4°C. Immunoreactive proteins were detected with a horse radish peroxidase-conjugated secondary antibody (Biorad Laboratories; 1:2,000–1:3,000 diluted in 2% milk powder in TBS-Tween) and an Enhanced chemiluminescence detection kit (Thermo Fisher Scientific, Waltham, MA, USA).

### Statistical Analysis

Data is presented as mean ± standard deviation (SD) or as boxplots, representing the mean (central black line), the upper and lower quartile (bottom and top boundaries of the box), and the largest and lowest values in the dataset that are not outliers (whiskers). Differences were tested for statistical significance using a Mann–Whitney *U* test (GraphPad Prism 6) at an α level of 0.05. Quantitative data were always obtained from at least three different mice.

## Results

### The Monoclonal Anti-CaMKII-β Antibody CB-β-1 Detected a Protein in Addition to CaMKII-β

**Figure 1:**
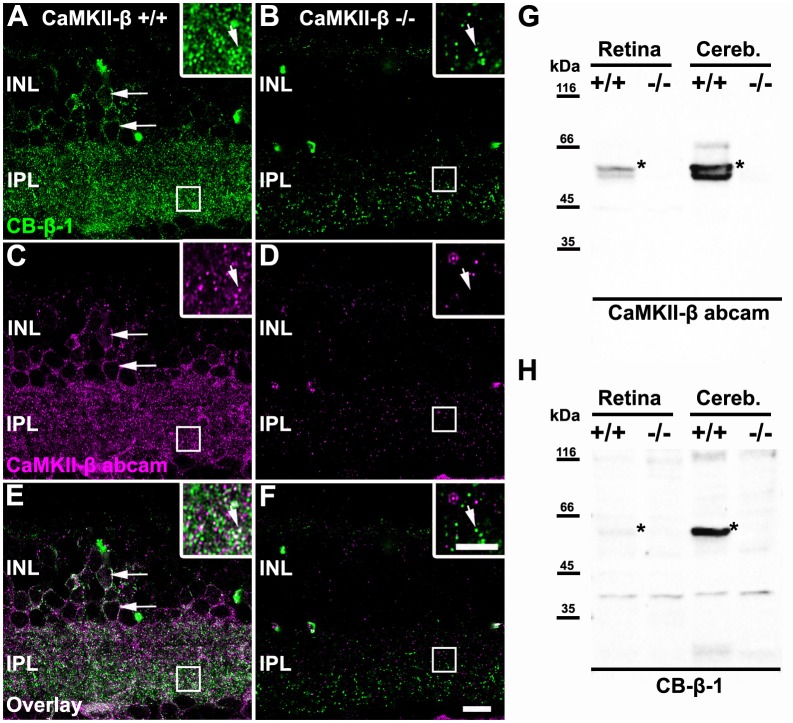
Monoclonal anti-Ca^2+^/calmodulin-dependent protein kinase II (CaMKII)-β antibodies (CB-β-1) detected CaMKII-β but also an additional protein in the retina. **(A–F)** Immunolabeling for CaMKII-β with two different antibodies, a monoclonal from Thermo Fisher Scientific, Waltham, MA, USA (CB-β-1, **A,B,E,F**) and a polyclonal from abcam (CaMKII-β abcam; **C–F**) in wild-type **(A,C,E)** and CaMKII-β-deficient mice** (B,D,F)**. Punctate staining remained in CaMKII-β-deficient animals when using the CB-β-1 antibody but was almost completely gone with the abcam antibody. Both antibodies showed substantial overlap in cell bodies (long arrows in **A,C,E**) but only weak overlap in the inner plexiform layer (IPL, small arrows in inset of **A,C,E**). Scale: 10 μm.** (G,H)** Western blots revealed that the anti-CaMKII-β antibody from abcam **(G)** recognized two different bands (asterisks) in wild-type retina which were not detected in CaMKII-β knockout tissue, confirming the specificity of the abcam antibody. In contrast, CB-β-1 antibodies recognized only a single band for CaMKII-β (asterisks) but several bands appeared which also occurred in CaMKII-β knockout tissue.

In the mammalian retina, CaMKII has been shown to increase homologous coupling between AII amacrine cells (Kothmann et al., 2012). A recent study in our lab suggests that this effect may be mediated by a particular CaMKII isoform: CaMKII-δ (Tetenborg et al., 2017). Our experiments further revealed colocalization of Cx36 and a second CaMKII isoform (CaMKII-β), suggesting that CaMKII and Cx36 interact in an additional unidentified retinal neuron. Therefore, we set out to determine the localization of CaMKII-β at electrical synapses in the inner retina. First, we tested the specificity of two commercially available antibodies for CaMKII-β, a monoclonal from Thermo Fisher Scientific, Waltham, MA, USA (CB-β-1) and a polyclonal from abcam (34703, Table 1) on CaMKII-β knockout retinas. In wild-type retinas, both antibodies displayed a punctate staining in the IPL and labeled the outlines of somata in the INL (arrows Figures 1A,C,E). However, in CaMKII-β knockout retinas, the punctate CB-β-1 labeling in the IPL partially remained (Figures 1B,F), indicating cross-reactivity with an unknown protein. In contrast, the immunoreactivity of the polyclonal antibody (abcam) almost completely vanished (Figure 1D), suggesting that this antibody is specific for CaMKII-β.

**Table 1:** List of antibodies used in this study.

Antibody	Host, type	Dilution	Source Cat. (No.)
CaMKII-β, clone: CB-β-1	Mouse, monoclonal	1:500	Thermo Fisher Scientific, 13-9800
CaMKII-β	Rabbit, polyclonal	1:1,000, 1:500 (WM)	abcam, 34703
Cx36, clone: 1E5H5	Mouse, monoclonal	1:500, 1:250 (WM)	Thermo Fisher Scientific, 37-4600
Cx36	Rabbit, polyclonal	1:500	Thermo Fisher Scientific, 36-4600
CtBP2	Mouse, monoclonal	1:500, 1:100 (WM)	BD Transduction Laboratories, 612044
HCN1, Clone: N70/28	Mouse, monoclonal	1:100–200	NeuroMab, 75-110

*WM gives the concentration used in retinal whole-mount stainings*.

These results were confirmed with western blot analysis comparing retina and cerebellar tissue from wild-type and CaMKII-β-deficient mice (Figures 1G,H). The polyclonal antibody from abcam recognized two proteins in retina and three proteins in cerebellar samples with molecular masses around the predicted sizes (49–60 kDa) for CaMKII-β splice variants (Wang et al., 2000). These bands were not detected in knockout tissue, confirming the specificity of the abcam antibody (Figure 1G). In contrast and consistent with the immunohistochemical stainings, the CB-β-1 antibody showed only a single band for CaMKII-β but also prominent bands which persisted in CaMKII-β-deficient tissue (Figure 1H), suggesting that this antibody recognizes an additional protein.

### CB-β-1 Antibodies Cross-Reacted With Cx36 and Recognized Its C-Terminal Tail

**Figure 2:**
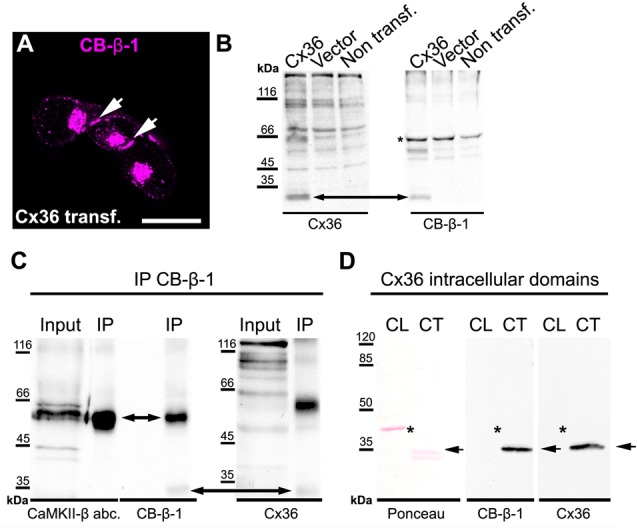
The anti-CaMKII-β antibody CB-β-1 cross-reacted with the C-terminal tail of connexin36 (Cx36).** (A,B)** Expression of Cx36 in N2A cells. CB-β-1 antibodies recognized Cx36 in transfected N2A cells in immunocytochemical stainings (**A**, arrows) and western blot experiments (**B**, arrow). It probably also recognized endogenous CaMKII-β expressed in N2A cells (asterisk). **(C)** Immunoprecipitation (IP) from retinal lysates using CB-β-1 antibodies revealed their binding to CaMKII-β and Cx36 whereas the CaMKII-β antibody from abcam (abc.) only detected CaMKII-β, as expected. **(D)** Binding of CB-β-1 antibodies to the purified C-terminal tail of Cx36 (arrow). Asterisks denote the expected band for the cytoplasmic loop, which was not detected by CB-β-1 and Cx36 antibodies. Scale: 20 μm.

The remaining CB-β-1 immunoreactivity in CaMKII-β-deficient retinas resembled the appearance of Cx36 in confocal scans in terms of puncta size and density in the proximal IPL. This result raised the question whether CB-β-1 actually recognized Cx36. To address this, we transfected N2A cells with Cx36 and Cx36-EGFP and tested for binding of CB-β-1 using immunocytochemistry and western blot (Figure 2). Indeed, the CB-β-1 antibody labeled gap junctions in Cx36 transfectants (Figure 2A, arrows). Cx36 and CB-β-1 antibodies detected the same band in Cx36-transfected N2A cells (Figure 2B, arrow), which was not present in lysates from untransfected and empty vector-transfected cells.

To further investigate the cross-reactivity of CB-β-1 antibodies on retinal tissue, we cross-linked CB-β-1 antibodies to magnetic protein G beads and performed an IP. We found that CB-β-1 antibodies precipitated two proteins: CaMKII-β (Figure 2C, short arrow, also detected with the polyclonal anti-CaMKII-β antibody from abcam) and—though very weakly—Cx36 (Figure 2C, long arrow, also detected with monoclonal antibodies for Cx36). These results were consistent with the labeling we observed in transfected N2A cells and the retina. Thus, we conclude that the CB-β-1 antibody recognizes CaMKII-β but also cross-reacts with Cx36.

To provide the final proof and also determine the epitope for CB-β-1 binding on Cx36, we generated and purified the cytoplasmic loop and C-terminal tail of Cx36 and fused them to a GST-protein. Then, we tested for CB-β-1 binding. Indeed, the CB-β-1 antibody recognized the C-terminal tail (Figure 2D, arrow) but not the cytoplasmic loop of Cx36 (Figure 2D, asterisks). The CB-β-1 antibody showed the same binding pattern as the monoclonal Cx36 antibody, which was used as positive control because it is known to bind to the C-terminal tail of Cx36 (Figure 2D; Hilgen et al., 2011). Taken together, this data confirmed that the monoclonal CaMKII-β antibody CB-β-1 specifically recognized Cx36 and bound to its C-terminal tail in retinal tissue.

### Colocalization of CaMKII-β and Cx36 Was Predominantly Detected in the Middle of the IPL

**Figure 3:**
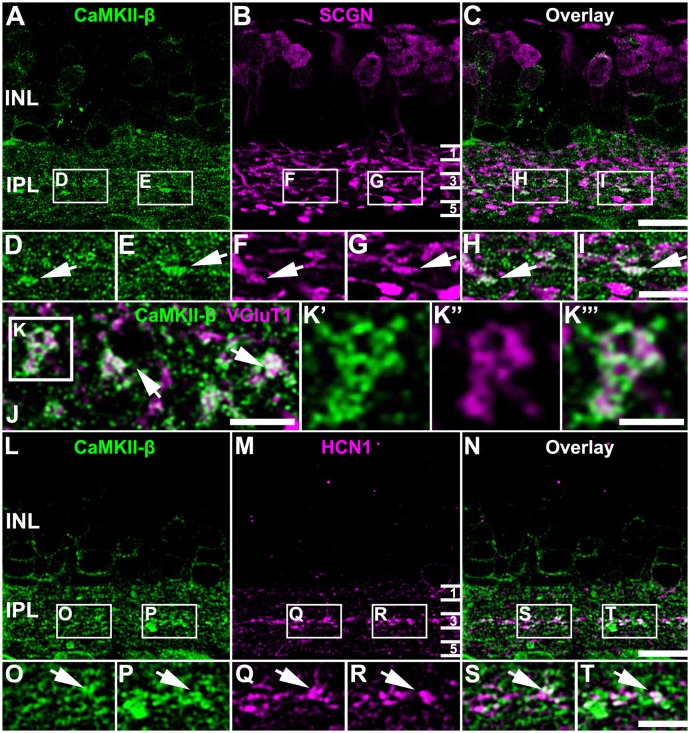
Association of CaMKII-β with terminals of type 5 bipolar cells.** (A–I)** CaMKII-β labeling (abcam antibody) was especially strong in an SCGN-positive bipolar cell type that stratified in the middle of the IPL. Weaker colocalization was also on bipolar cell terminals that were located more proximally, and likely represent type 6 ON bipolar cells. **(J–K”’)** A retinal whole-mount, labeled for CaMKII-β and VGluT1, was scanned in the middle of the IPL. VGluT1-labeled bipolar cell terminals were prominently decorated with CaMKII-β immunoreactive puncta.** (L–T)** CaMKII-β immunoreactivity was tightly associated with terminals of type 5 bipolar cell terminals, which were labeled with HCN1. Scale: **(A–C,L–N)**, 10 μm; **(D–I,J,K’–K”’,O–T)**, 5 μm.

To determine the synaptic localization of CaMKII-β in the IPL of the mouse retina, we used only the polyclonal antibody for CaMKII-β from abcam. We double-labeled retinas for secretagogin (SCGN), a marker for a subset of cone bipolar cells (Puthussery et al., 2010). CaMKII-β labeling was detected in the entire IPL but was very strongly expressed in bipolar cell terminals that stratified in the middle layer of the IPL (Figures 3A–I). This was confirmed by labeling retinal whole-mounts for CaMKII-β and VGluT1, a marker for all bipolar cell terminals. In the middle of the IPL, strong CaMKII-β labeling was detected in and surrounding bipolar cell terminals (Figures 3J–K”’, arrows). Moreover, the strong CaMKII-β labeling in that region partially colocalized with HCN1 (Figures 3L–T), a marker for type 5 bipolar cells (Müller et al., 2003; Hellmer et al., 2016), suggesting that CaMKII-β expression is strong in type 5 bipolar cells. These bipolar cells form four subtypes (Tsukamoto and Omi, 2017) but because subtype-specific markers were not available, we could not ascribe this strong CaMKII-β expression to a particular subtype of type 5 bipolar cells. Please note that colocalization with SCGN was also found for bipolar cell terminals in the proximal IPL, which presumably represent type 6 bipolar cells.

**Figure 4:**
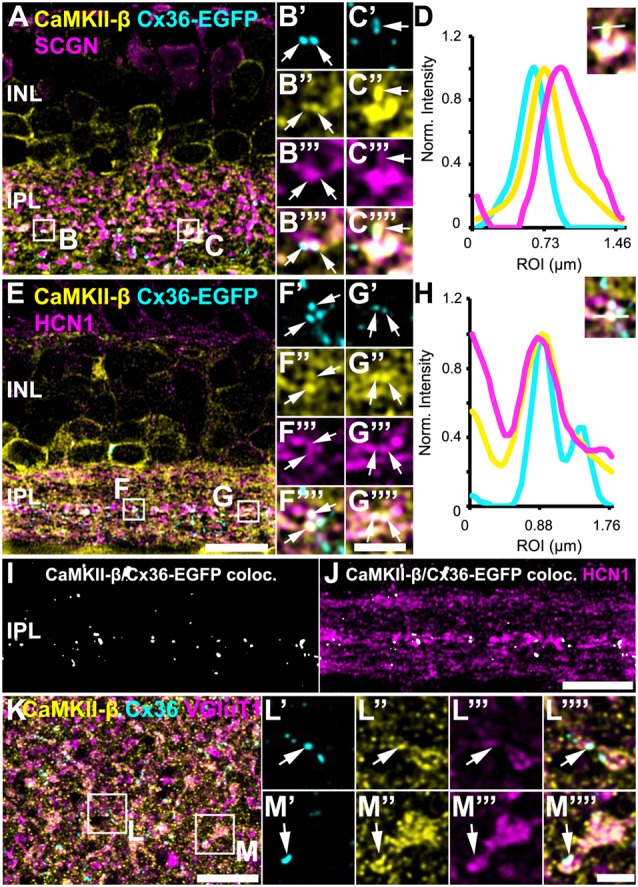
Colocalization of CaMKII-β and Cx36/Cx36-EGFP on terminals of type 5 bipolar cells. **(A–C””)** Cx36-EGFP and CaMKII-β immunoreactivity (abcam antibody) mainly colocalized on SCGN-labeled terminals of bipolar cells. **(D)** A line scan revealed close proximity of Cx36-EGFP and CaMKII-β on bipolar cell terminals. The line scan was taken from the area marked as **(C)**. **(E–G””)**. Triple stainings with HCN1 revealed that Cx36-EGFP and CaMKII-β mainly colocalized on terminals of type 5 bipolar cell terminals, stratifying in sublamina 3. **(H)** Line scan of all three channels shown in **(G)**. **(I)** Maximum projection of the highlighted Cx36-EGFP/CaMKII-β colocalization. **(J)** Merged single scans of highlighted Cx36-EGFP/CaMKII-β colocalization and HCN1. **(K)** Maximum projection (32 slices) of a retinal whole-mount, triple-labeled with CaMKII-β, Cx36 and VGluT1. **(L–M””)** Magnified bipolar cell terminals revealed Cx36 and CaMKII-β colocalization. Scale: **(A,E,I–K)**, 10 μm; **(B’–C””,F’–G””,L’–M””)**, 2.5 μm.

In Tetenborg et al. (2017), we identified colocalization of CaMKII-β and the gap junction protein Cx36 in a region that corresponded to the third sublamina of the IPL. Therefore, we tested for CaMKII-β/Cx36 colocalization on type 5 bipolar cells and triple-labeled retinas for Cx36, CaMKII-β and either SCGN (Figures 4A–D) or HCN1 (Figures 4E–J). These stainings revealed substantial colocalization of Cx36 and CaMKII-β on type 5 bipolar cell terminals (arrows in Figures 4B’–C””, F’–G””). Line scans that were obtained from individual gap junction plaques confirmed the close association of Cx36, CaMKII-β and the respective bipolar cell marker (Figures 4D,H). A maximum projection of highlighted Cx36/CaMKII-β colocalization demonstrates that puncta were mainly confined to the middle of the IPL (Figure 4I). Merging a single scan of this binary image with HCN1, it became visible that colocalized puncta were predominantly situated on the terminals of HCN1-positive type 5 bipolar cells (Figure 4J).

Retinal whole-mounts labeled for CaMKII-β, Cx36 and VGluT1 (a marker for all bipolar cell terminals), gave similar results: in the middle of the IPL, CaMKII-β immunolabeling almost filled the VGluT1-positive terminals and partially colocalized with Cx36-immunoreactive puncta (Figures 4K–M””, arrows). In addition, quantification analysis on Cx36-EGFP-positive whole-mount retinas revealed that 24 ± 2% of all Cx36-EGFP-immunoreactive puncta in the middle of the IPL colocalized with CaMKII-β, whereas only 6 ± 1% of all CaMKII-β-immunoreactive structures colocalized with Cx36-EGFP (Table 2; data are given as mean ± SD, from three mice).

**Table 2:** Quantification of CaMKII-β/Cx36 colocalization in the middle of the IPL.

	Cx36	CaMKII	Cx36/CaMKII	CtBP2	Cx36/CaMKII/CtBP2
Overall puncta	553 ± 98	2,362 ± 101		2,194 ± 409
Colocalized puncta			130 ± 12		7 ± 3
% colocalization	24 ± 2	6 ± 1		<1	1 ± 1
No. of mice	3	3	3	3	3

*CaMKII denotes CaMKII-β. Values are given as mean ± SD*.

Please note that the middle of the IPL also contained colocalized Cx36 and CaMKII-β immunoreactivity that was not associated with bipolar cell terminals. These puncta can probably be assigned to gap junctions on amacrine or ganglion cells.

Taken together, our data imply that CaMKII-β and Cx36 colocalize at type 5 bipolar cell terminals and presumably to a smaller degree on type 6 bipolar cells. However, as only a small fraction of the CaMKII-β immunoreactivity contributes to this colocalization, our data suggest that CaMKII-β may fulfill several functions at bipolar cell synapses.

### CaMKII-β and Cx36 Were Closely Associated With Synaptic Ribbons on Bipolar Cell Terminals

**Figure 5:**
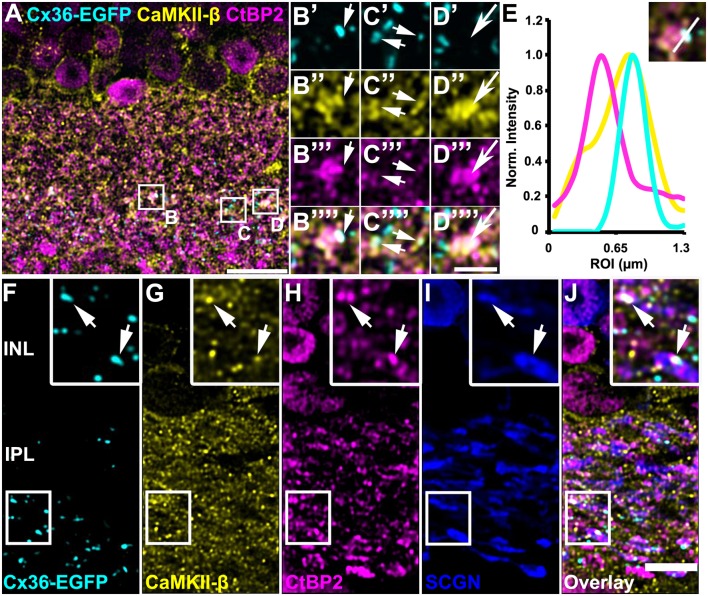
CaMKII-β immunoreactivity and Cx36-EGFP were closely associated with synaptic ribbons on bipolar cell terminals.** (A–D””)** Triple stainings revealed close proximity of CaMKII-β (abcam antibody), Cx36-EGFP and CtBP2 on SCGN-positive bipolar cell terminals. **(E)** Line scans of the individual gap junction plaque shown in **(B)**. **(F–J)** Cx36-EGFP expression and triple labeling for CaMKII-β, CtBP2 and SCGN. Scale: **(A)**, 10 μm; **(B–D””)**, 2.5 μm; **(F–J)**, 5 μm.

Kothmann et al. (2012) showed that gap junctions in AII amacrine cells are modulated *via* non-synaptic NMDA receptors that are activated by glutamate spillover from a neighboring chemical synapse. Our observation, however, might point to a different mechanism for CaMKII signaling as we found most Cx36/CaMKII-β puncta directly inside bipolar cell terminals. This localization suggests that Cx36 and CaMKII-β may be located in close vicinity to the glutamate release site. To confirm this hypothesis, we additionally labeled for ribbon synapses with antibodies directed against the C-terminal binding protein 2 (CtBP2). We found Cx36 and CaMKII-β to be marginally colocalized with synaptic ribbons because less than 1% of all CtBP2 puncta showed additional Cx36 and CaMKII-β labeling (Figures 5A–E, short arrows; Table 2). However, we did find that many Cx36/CaMKII-β-immunoreactive puncta were in close association with synaptic ribbons in type 5 bipolar cells, as was revealed by additional labeling for SCGN (Figures 5F–J, arrows in insets). Thus, despite the lack of colocalization, the close association of Cx36, CaMKII-β and glutamatergic synapses could allow for activity-dependent potentiation of electrical synapses in bipolar cells.

### Do Cx36/CaMKII-β-Colocalized Puncta Represent Contacts to AII and A8 Amacrine Cells?

**Figure 6:**
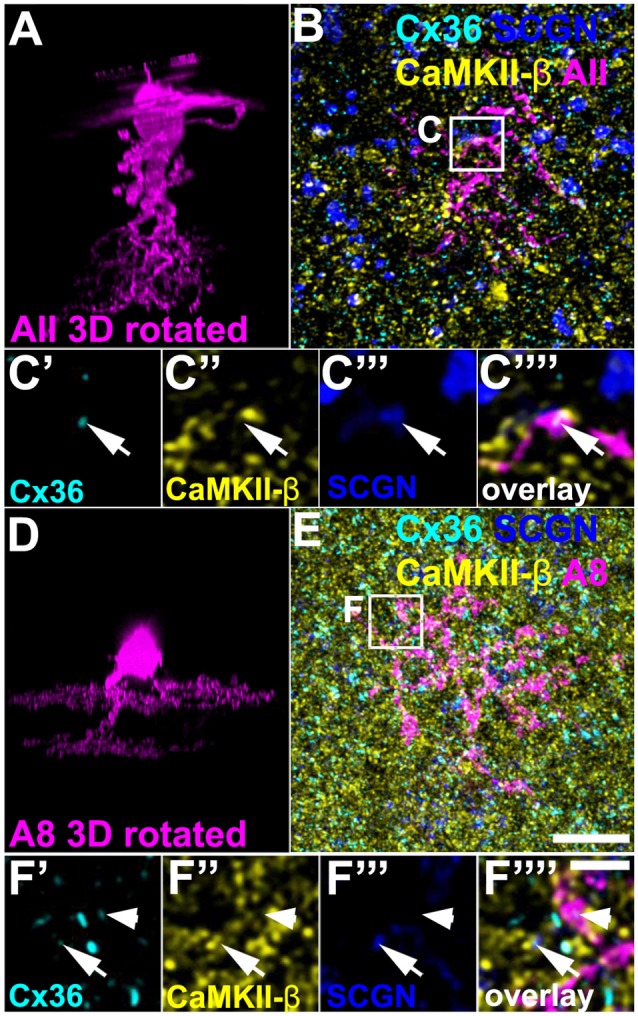
CaMKII-β was absent from gap junctions that AII and A8 amacrine cells form with bipolar cells.** (A)** XZ rotation of an Alexa568-injected AII amacrine cell. Please note that the “whirl” around the soma represents an injection artifact. **(B)** Maximum projection (20 optical sections) of the injected cell, labeled for CaMKII-β, Cx36, and SCGN.** (C’–C””)** Colocalization of Cx36 and SCGN was associated with processes of the injected AII amacrine cell but not with CaMKII-β immunoreactivity. **(D–F””)** Same as **(A–C””)** but showing an Alexa568-injected A8 amacrine cell. Colocalization of Cx36 and CaMKII-β (indicated by arrows) was not associated with processes of the injected A8 amacrine cell. Cx36 immunoreactivity on the A8 dendrite was not associated with CaMKII-β (arrowhead). Scale: **(A,B,D,E)**, 10 μm; **(C’–C””,F’–F””)**, 2.5 μm.

Above, we have shown that a low amount of CaMKII-β colocalized with Cx36 in type 5 (and presumably type 6) bipolar cell terminals. Next, we tried to determine the gap junctional partner at these sites. First, we tested for AII amacrine cells because these cells were shown to couple to type 5 and 6 bipolar cells (Veruki and Hartveit, 2002; Tsukamoto and Omi, 2017). We injected AII cells in C57BL/6J retinas with Alexa 568 and additionally labeled the whole-mounts for SCGN, CaMKII-β and Cx36. We found Cx36 to be present on the dendrites of the injected AII cell and opposed to SCGN-positive bipolar cell terminals (Figures 6A–C””, arrows). However, on AII dendrites Cx36 did not colocalize with CaMKII-β, indicating that Cx36/CaMKII-β-containing gap junctions are not present on AII cells.

In addition, we Alexa 568-injected A8 amacrine cells in the Ier5-GFP line because these cells are also coupled to ON bipolar cells in the mouse retina (Lee et al., 2015; Yadav et al., 2019). Cx36/CaMKII-β colocalization on SCGN-positive bipolar cell terminals occurred separated from dye-filled dendrites of A8 cells (Figures 6D–F””, arrows), suggesting that CaMKII-β does not regulate the coupling between A8 amacrine and ON bipolar cells. Conversely, these results imply that type 5 bipolar cells may make Cx36-containing gap junctions with an unknown cell type and these gap junctions may be regulated by CaMKII-β.

### CaMKII-β Deficiency Did Not Alter Cx36 Expression in Bipolar Cell Terminals

**Figure 7:**
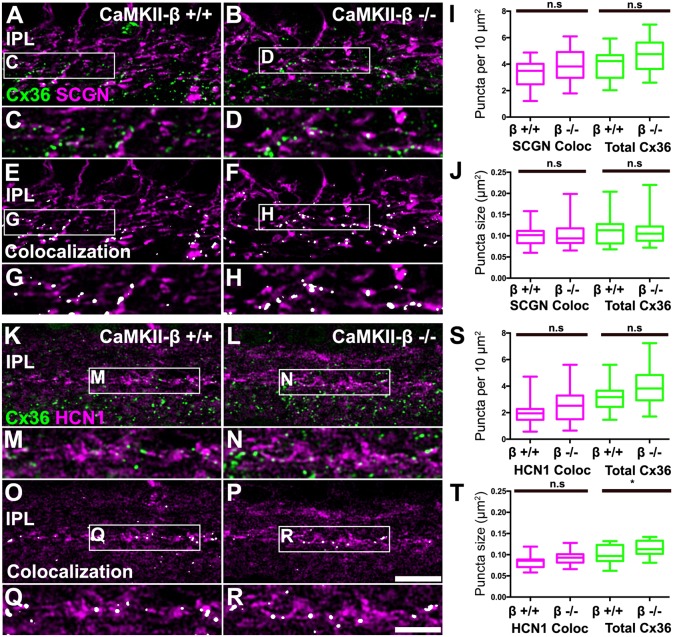
CaMKII-β deficiency did not affect puncta size and frequency of Cx36 clusters on bipolar cell terminals in the mid-IPL.** (A–H)** Comparison of Cx36 puncta expression on SCGN-positive bipolar cell terminals between WT and CaMKII-β KO retinas. Colocalized puncta shown in **(A–D)** are highlighted in white in **(E–H)**. **(I,J)** Quantitative analysis of Cx36-immunoreactive puncta colocalizing with SCGN. We did not find any significant differences between genotypes (Mann–Whitney *U* test, *n* = 22 ROIs from three mice for each genotype, *p* > 0.23 for all comparisons). **(K–R)** Testing for Cx36 immunoreactivity on HCN1-labeled bipolar cell terminals. Colocalized puncta are highlighted in white. **(S,T)** Analysis of colocalized puncta revealed no differences in cluster frequency and size between genotypes (Mann–Whitney *U* test, *n* = 21/23 ROIs from three mice for control/CaMKII-β-deficient mice, *p* > 0.0674 for all comparisons). Only a slight difference in the average size of all Cx36 puncta was detected (Mann–Whitney *U* test, *p* = 0.0247), which was not present in double stainings with SCGN **(J)**. n.s. denotes non-significant; *p < 0.05. Scale: **(A,B,E,F,K,L,O,P)**, 10 μm; **(C,D,G,H,M,N,Q,R)**, 5 μm.

A recent study implies that CaMKII-β but not CaMKII-α is essential for electrical synapse formation in the inferior olive (Bazzigaluppi et al., 2017), contrasting previous results from Kothmann et al. (2012) who showed that CaMKII inhibition disrupts Cx36 phosphorylation but not the formation of Cx36-containing gap junctions. Our observations so far indicated that CaMKII-β expression occurred at gap junctions in type 5 bipolar cells, which raised the question whether this isoform fulfills a similar function as in the inferior olive and mediates gap junction formation in these neurons. We tested this hypothesis and determined the size and number of Cx36 puncta on SCGN- (Figures 7A–J) and HCN1-positive bipolar cells (Figures 7K–T) of control and CaMKII-β-deficient retinas. To estimate Cx36 puncta expressed in type 5 bipolar cells, we placed a ROI in the middle of the IPL. However, the total number and size of Cx36 were almost unchanged, as was the degree of colocalization with bipolar cell markers (Figures 7I,J,S,T). The only significant difference (*p* = 0.0247) we found was in the size of the Cx36 puncta (Figure 7T) in samples that were also labeled for HCN1. But given the lack of a difference on SCGN-labeled terminals (Figure 7J) the significance of this finding is unclear. Our analysis led us to conclude that CaMKII-β is not necessary for the formation of electrical synapses in the terminals of type 5 bipolar cells.

## Discussion

### Interaction of CaMKII-β and Cx36 on Type 5 Bipolar Cell Terminals

Although CaMKII is considered as a key molecule of synaptic plasticity, the role of its different isoforms in modulating electrical synapses remains unknown. Our lab recently demonstrated that among the four CaMKII isoforms, only the β- and -δ subunits associate with gap junctions in the retina (Tetenborg et al., 2017). Interestingly, CaMKII-β and CaMKII-δ are differentially associated with gap junctions in the murine retina: CaMKII-β is predominantly found in the outer retina, whereas CaMKII-δ is associated with Cx36 in AII and TH2 amacrine cells. Likewise, the localization with respect to glutamatergic synapses differs between the two isoforms: CaMKII-β is strongly expressed postsynaptically to rod bipolar cell terminals, whereas CaMKII-δ is found close to photoreceptor synaptic ribbons and inside rod bipolar cell terminals. Here, we demonstrate that the terminals of HCN1-positive type 5 bipolar cells are an exception to this pattern; they expressed large amounts of CaMKII-β *inside* the terminal where it partially colocalized with Cx36 and CtBP2. This suggests that: (1) CaMKII-β may have a similar function for type 5 cells as CaMKII-δ for rod bipolar cells; and (2) CaMKII subunit composition at gap junctions is cell type-specific although we cannot entirely exclude that the two CaMKII isoforms are expressed in overlapping cell populations.

Some of the four subtypes of type 5 bipolar cells are known to form gap junctions with AII amacrine cells (Tsukamoto and Omi, 2017). This is also true for type 6 bipolar cells, which additionally couple to A8 amacrine cells (Yadav et al., 2019). However, the Cx36/CaMKII-colocalized puncta, associated with type 5 (and to a lesser degree type 6) bipolar cell terminals, were not located on AII or A8 amacrine cells, ruling out both small-field amacrine cells as coupling partners. Thus, it is intriguing to speculate that there is at least one additional synaptic partner coupled to type 5 cells. As type 5 cells also show across-subtype coupling (Tsukamoto and Omi, 2017), CaMKII-β may regulate the coupling between bipolar cells.

However, as dye injections into bipolar cells are very difficult, we could not study inter-bipolar cell coupling systematically. But in one instance, when we dye-injected a type 5 bipolar cell, we detected Cx36 colocalized with CaMKII-β at a contact point of two bipolar cell dendrites in layer 3 of the IPL (Supplementary Figure S1). This may represent heterologous coupling between different type 5 bipolar cell subtypes, as was reported before (Tsukamoto and Omi, 2017).

Neuronal gap junctions are not as static as previously thought and the efficacy of these synapses can be dynamically modulated by interaction with glutamatergic synapses (reviewed in Pereda, 2014). In the retina, CaMKII was shown to mediate a signaling cascade that changes the phosphorylation state of Cx36 in AII amacrine cells (Kothmann et al., 2012). The activation of CaMKII is initiated by Ca^2+^ entry *via* extra-synaptic NMDA receptors. Here, we found CaMKII-β and Cx36 colocalization on bipolar cell terminals. Although CaMKII-β/Cx36-colocalized structures did not colocalize with CtBP2, the ribbon marker, the close association with the glutamate release site may allow for activity-dependent plasticity and crosstalk between electrical and chemical synapses in the same bipolar cell terminal.

### CaMKII-β and Electrical Synapse Formation

Our data indicate that CaMKII-β is not required for electrical synapse formation in the mouse retina because genetic deletion of this subunit had no effect on the number and size of Cx36-immunoreactive puncta on bipolar cell terminals. This was also corroborated by the fact that only a subset of Cx36-containing gap junctions colocalized with CaMKII-β in the mouse retina (this study; Tetenborg et al., 2017). In contrast, Cx36 and CaMKII were reported to strongly overlap in the murine inferior olive (Alev et al., 2008); and Bazzigaluppi et al. (2017) showed that genetic deletion of CaMKII-β reduces the density of Cx36 immunoreactivity in this brain area. Thus, CaMKII-β presumably fulfills a critical function in gap junction stability in one part of the CNS (inferior olive) but not in another (retina). As genetic ablation of CaMKII-β did not induce any alterations in gap junction size and frequency, we assume that the role of this subunit for retinal gap junctions is to modulate channel gating but not gap junction formation or maintenance.

### What Does Cause the Cross-Reaction of CB-β-1 With Cx36?

One interesting aspect of this study was that we uncovered a cross-reaction of CB-β-1 with the C-terminal tail of Cx36. This was completely unexpected as the antibody has been widely used in a variety of different publications (Liu and Cooper, 1996; Brigman et al., 2010; Kool et al., 2016). Presumably, this cross-reaction was overseen because most research on CaMKII focuses on chemical synapses and long-term potentiation, and no other lab so far investigated distinct CaMKII subunits in relation to electrical synapses. Moreover, Cx36 tends to dimerize when the protein samples are boiled prior to gel electrophoresis. This often results in a western blot band of similar height than the CaMKII-β band, making confusions likely.

Still, it is intriguing that CaMKII-β and Cx36 do interact and can be recognized by the same antibody although both molecules belong to completely different protein families, serine/threonine kinases and ion channels, respectively. The epitope of CB-β-1 is unknown as the entire CaMKII-β protein was initially used for immunization, allowing us to only hypothesize what actually may have caused this cross-reaction. A previous study from Alev et al. (2008) reported that CaMKII interacts with Cx36 in a similar way as it does with glutamate receptors. This study further indicated a sequence similarity between the C-terminal tail of Cx36 and the auto-inhibitory region of CaMKII. If this very region represented the epitope for the monoclonal CB-β-1 antibodies, its sequence similarity to Cx36 could explain the cross-reactivity we observed. Consistent with this hypothesis, CB-β-1 antibodies recognized the isolated C-terminal tail but not cytoplasmic loop of Cx36. However, the amino acid sequence in question is also present in CaMKII-α but we did not observe any cross-reactivity with the α-subunit in retinal sections (personal observation S. Tetenborg; see also Tetenborg et al., 2017), although minor binding of CB-β-1 to CaMKII-α is known from western blots (personal observation S. Tetenborg; see also Thermo Fisher Scientific, Waltham, MA, USA, data sheet). At the very least, this finding once more confirms that knockout tissue is essential to definitely test the specificity of an antibody.

In summary, our data adds to the growing body of evidence that electrical and chemical synapses share many features (here: modulation by CaMKII-β). The differential and distinct expression pattern of CaMKII isoforms may provide the molecular basis for the fine-tuning of glutamate release and gap junctional coupling in retinal bipolar cells. It may even represent a substrate for extensive crosstalk between the two means of synaptic transmission.

## Data Availability

The datasets generated for this study are available on request to the corresponding author.

## Ethics Statement

All procedures were approved by the local animal care committee (Niedersaechsisches Landesamt fuer Verbraucherschutz und Lebensmittelsicherheit) and were in compliance with the guidelines for the welfare of experimental animals issued by the European Communities Council Directive of 24 November 1986 (86/609/EEC) and the laws of the Federal Government of Germany (Tierschutzgesetz; BGBl. I S. 1206, 1313 and BGBl. I S. 1934).

## Author Contributions

KD, ST and UJ-B designed experiments. ST, SY and BB performed experiments. ST prepared all figures (revised by KD). ST wrote a first draft of the manuscript which was revised by KD. All contributed to the interpretation of data. All authors edited and commented on the manuscript and KD finalized it.

## Conflict of Interest Statement

The authors declare that the research was conducted in the absence of any commercial or financial relationships that could be construed as a potential conflict of interest.

## Footnotes

^1^https://fiji.sc/
