# Supplementary material for: Localization of Retinal Ca2+/Calmodulin-Dependent Kinase II-β (CaMKII-β) at Bipolar Cell Gap Junctions and Cross-Reactivity of a Monoclonal Anti-CaMKII-β Antibody With Connexin36
Source: Front Mol Neurosci. 2019 Aug 28;12:206. doi: 10.3389/fnmol.2019.00206 (PMC6724749; doi:10.3389/fnmol.2019.00206)
Supplement: Supplementary file 1 [file Data_Sheet_1.pdf]

Localization of Retinal Ca^{2+}/Calmodulin-dependent Kinase II-β (CaMKII-β) at Bipolar Cell Gap Junctions and Cross-reactivity of a Monoclonal Anti-CaMKII-β Antibody with Connexin36

Stephan Tetenborg[1], Shubhash Chandra Yadav[1], Bianca Brüggen[1#], Georg R. Zoidl[2], Sheriar G. Hormuzdi[3], Hannah Monyer[4], Geeske M. van Woerden[5], Ulrike Janssen-Bienhold[6,7], Karin Dedek[1,7*]

[1]Animal Navigation/Neurosensorics, Institute for Biology and Environmental Sciences, University of Oldenburg, Oldenburg, Germany
[2]Department of Biology & Center for Vision Research, York University, Toronto, Canada
[3]Division of Neuroscience, Ninewells Hospital and Medical School, University of Dundee, Dundee, UK
[4]Cancer Research Center (DKFZ), Heidelberg, Germany
[5]Department of Neuroscience, Erasmus MC University Medical Center, Rotterdam, Netherlands
[6]Visual Neuroscience, Dept. of Neuroscience, University of Oldenburg, Oldenburg, Germany
[7]Research Center Neurosensory Science, University of Oldenburg, Oldenburg, Germany
#Present address: Institute for Anatomy and Molecular Neurobiology, Westfälische Wilhelms-University, Münster, Germany

Running title: CaMKII-β and retinal gap junctions

***Correspondence:**
Karin Dedek
karin.dedek@uni-oldenburg.de

1 Supplementary Figures and Tables

1.1 Supplementary Figures

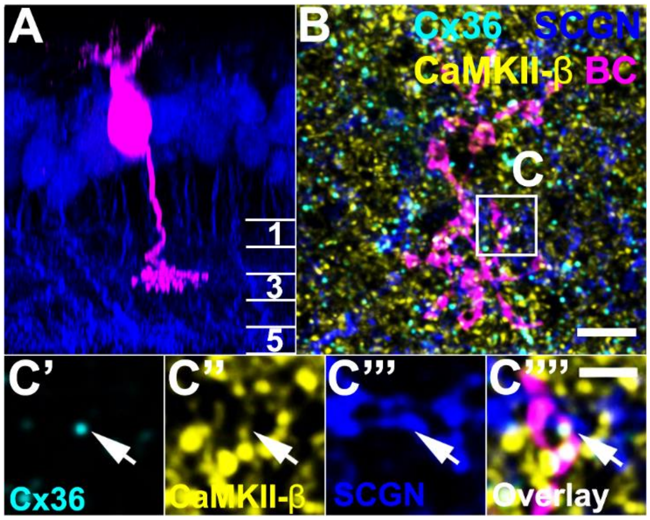

Supplementary Figure 1. CaMKII-β may regulate inter-bipolar cell gap junctions. **(A)** XZ rotation of an Alexa568-injected type 5 bipolar cell (BC), immunolabeled for secretagogin (SCGN). Numbers and lines indicate the different layers of the inner plexiform layer. **(B)** XY view, showing the axon terminal of the same cell in the retinal whole-mount, labeled for connexin36 (Cx36), CaMKII-β and SCGN. The area marked by the white square is shown in higher magnification as single confocal scan in C'-C''''. **(C'-C'''')** Cx36 colocalized with CaMKII-β at a contact point of two bipolar cell dendrites in layer 3 of the inner plexiform layer. It presumably represents heterologous coupling between different type 5 bipolar cell subtypes (Tsukamoto and Omi, 2017). Scale bar: B, 5 µm, C'-C'''', 2 µm.

2 Supplementary References

Tsukamoto, Y., and Omi, N. (2017). Classification of Mouse Retinal Bipolar Cells: Type-Specific Connectivity with Special Reference to Rod-Driven AII Amacrine Pathways. *Front Neuroanat* 11, 92. doi:10.3389/fnana.2017.00092.
